# Global Transcriptome and Physiological Responses of *Acinetobacter oleivorans* DR1 Exposed to Distinct Classes of Antibiotics

**DOI:** 10.1371/journal.pone.0110215

**Published:** 2014-10-17

**Authors:** Aram Heo, Hyun-Jin Jang, Jung-Suk Sung, Woojun Park

**Affiliations:** 1 Laboratory of Molecular Environmental Microbiology, Department of Environmental Science and Ecological Engineering, Korea University, Seoul, Republic of Korea; 2 Department of Life Science, Dongguk University, Seoul, Republic of Korea; University of Illinois at Chicago College of Medicine, United States of America

## Abstract

The effects of antibiotics on environment-originated nonpathogenic *Acinetobacter* species have been poorly explored. To understand the antibiotic-resistance mechanisms that function in nonpathogenic *Acinetobacter* species, we used an RNA-sequencing (RNA-seq) technique to perform global gene-expression profiling of soil-borne *Acinetobacter oleivorans* DR1 after exposing the bacteria to 4 classes of antibiotics (ampicillin, Amp; kanamycin, Km; tetracycline, Tc; norfloxacin, Nor). Interestingly, the well-known two global regulators, the *soxR* and the *rpoE* genes are present among 41 commonly upregulated genes under all 4 antibiotic-treatment conditions. We speculate that these common genes are essential for antibiotic resistance in DR1. Treatment with the 4 antibiotics produced diverse physiological and phenotypic changes. Km treatment induced the most dramatic phenotypic changes. Examination of mutation frequency and DNA-repair capability demonstrated the induction of the SOS response in *Acinetobacter* especially under Nor treatment. Based on the RNA-seq analysis, the glyoxylate-bypass genes of the citrate cycle were specifically upregulated under Amp treatment. We also identified newly recognized non-coding small RNAs of the DR1 strain, which were also confirmed by Northern blot analysis. These results reveal that treatment with antibiotics of distinct classes differentially affected the gene expression and physiology of DR1 cells. This study expands our understanding of the molecular mechanisms of antibiotic-stress response of environment-originated bacteria and provides a basis for future investigations.

## Introduction

Antibiotics are abundant in various environmental habitats such as seawater, plants, sludge, and soils [1]–[3]. Because antibiotics affect our ecosystem, which includes the microbial diversity and abundance in the environment, they are widely considered to act as key pollutants [4], [5]. Although antibiotics contaminate the environment, how antibiotics affect environment-originated bacteria and their evolution remains poorly understood. Because most antibiotics used for treating infections are produced by environmental microorganisms, antibiotic resistance genes and mechanisms could exist in nonclinical habitats [6]. In natural environments, antibiotic production and resistance might be considered as biochemical warfare to eliminate competing organisms because antibiotics suppress bacterial growth and metabolism [7]. Antibiotics of distinct classes act on different targets through specific mechanisms: β-lactams lead to autolysis by interfering with cell-wall biosynthesis [8]; aminoglycosides cause mistranslation by targeting the 30S subunit of the ribosome [9], [10]; tetracycline inhibits protein synthesis by disrupting the binding of aminoacyl-tRNA to the mRNA-ribosome complex [11]; and fluoroquinolones inhibit DNA replication by binding with DNA gyrase and topoisomerase [12]. Antibiotic resistance could be acquired through several ways: i) the action of antimicrobial-inactivating enzymes, ii) reduced access of antimicrobials to bacterial targets (decreased outer-membrane permeability and overexpression of multidrug efflux pumps), and iii) mutations that change targets or cellular functions [13]. Many clinical and environmental bacteria have multiple antibiotic-resistance mechanisms [13].

The diesel-degrading *A. oleivorans* DR1 was isolated from the rice paddy soil and its genome was completely sequenced [14]. Our previous studies demonstrated that quorum sensing and biofilm formation are important for diesel-degradation in DR1 cells [14]. Most antibiotic resistance studies of *Acinetobacter* species have largely focused on pathogenic *Acinetobacter* such as *Acinetobacter baumannii* owing to high level of multidrug resistance. Transcriptional responses to various antibiotics and their regulation have not been extensively defined with *Acinetobacter* species. Reducing access to bacterial targets by means of decreasing permeability and using strong efflux systems has been reported as a major cause of multidrug resistance in *Acinetobacter* species [15]. Because the genome of DR1 is similar to those of the human pathogens *A. calcoaceticus* and *A. baumannii* [16], the DR1 strain is appropriate for studying antibiotic effects in evolutionary aspect. To identify key genes and their functions in the antibiotic resistance of environment-originated bacteria, we performed whole-transcriptome profiling of *Acinetobacter oleivorans* DR1 using RNA-Seq technique. with four representative antibiotics: ampicillin (Amp), kanamycin (Km), tetracycline (Tc), and norfloxacin (Nor).

Bacteria could exhibit physiological changes by changing global gene expression pattern in response to low concentration of antibiotics [17]. To promote understanding how antibiotic resistance develops in DR1, we also conducted several physiological tests on DR1 under distinct antibiotic stresses. Herein we provide both transcriptomic and experimental evidence of antibiotic-resistance mechanisms in DR1. Elucidating transcriptional and physiological responses to distinct antibiotics might establish novel molecular basis for antibiotic-resistance mechanisms of *Acinetobacter* species.

## Results

### Comparative transcriptome analysis of *A. oleivorans* DR1 exposed to sub-MICs of antibiotics of distinct classes

**Figure 1:**
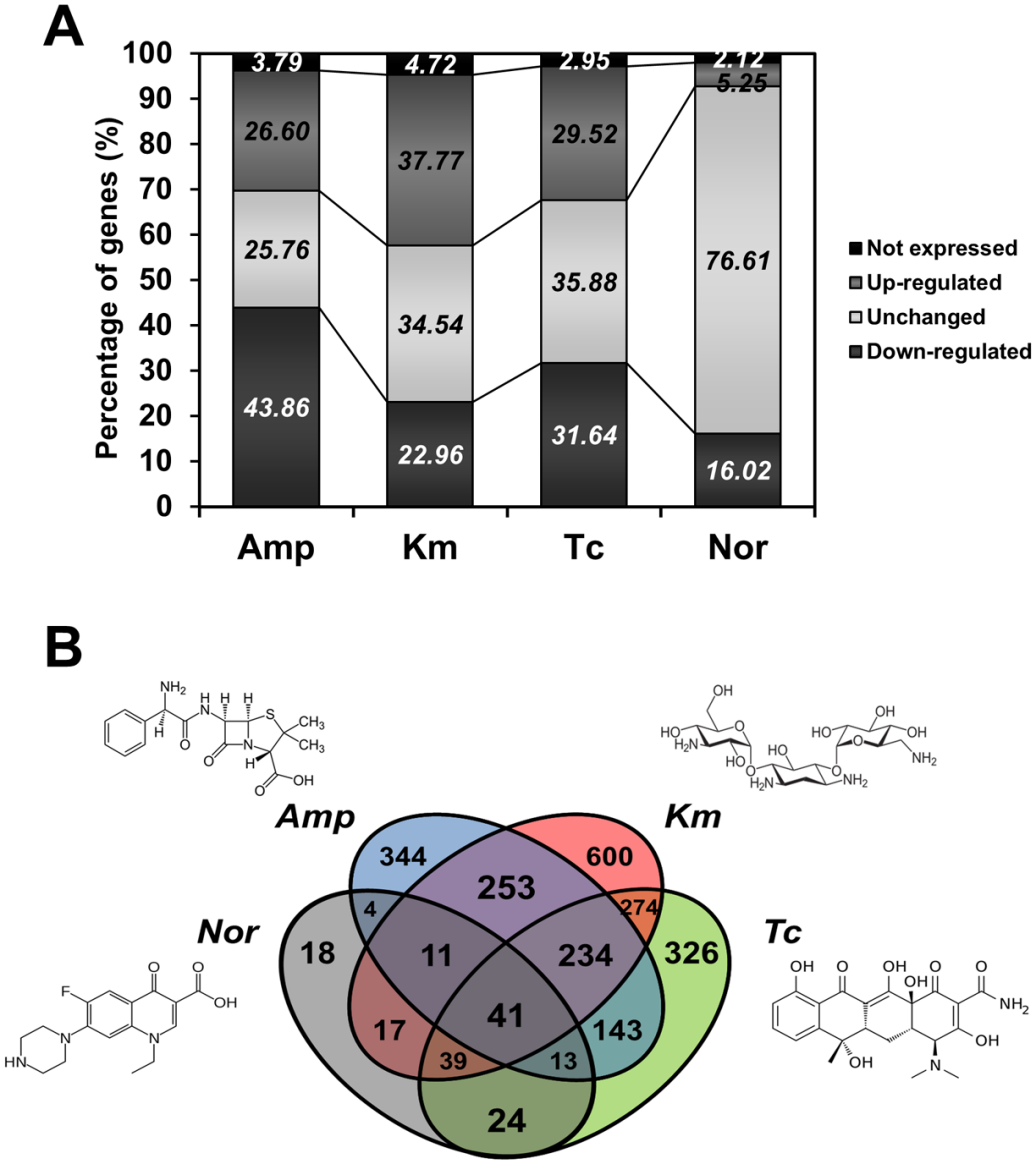
A summary of genes upregulated and downregulated by distinct classes of antibiotics. (A) The percentages of up- and down-regulated genes under treatment with 4 antibiotics. (B) Venn-diagram showing the number of overlapping genes upregulated by antibiotics of distinct classes. Fold-changes shown are a comparison of the RPKM values of exponentially growing control cells and of cells treated with each antibiotic. Upregulation of gene expression is >1.5-fold change in RPKM value, downregulation is <1.5-fold change.

Antibiotics have been reported to affect bacterial transcription in a concentration-dependent manner, and using antibiotics at concentrations as high as the MIC can cause extensive cellular stress and death [17]. To determine the appropriate concentration of antibiotics for the antibiotics induced transcriptome, we measured MICs of 4 classes of antibiotics in various cell densities (10^5^–10^8^ CFU/mL). When cell density increased, the MIC of antibiotics was increased (Figure S1). This result demonstrates the relationship between the cell density of bacteria and the MIC of antibiotics. Because of transcript modulation decreases at high antibiotics concentration, DR1 cells were exposed to sub-MIC of distinct antibiotic classes. Sub-MIC of antibiotic allows susceptible strains to grow, but induces stress responses. The highest MICs measured were for Amp (100–200 µg/mL), and by comparison, DR1 cells were more susceptible to other antibiotics (MICs, 1–8 µg/mL). We speculate that high number of lactamases encoded by the DR1 genome confer high resistance to Amp (and thus the high MIC ranges). In this study, we selected the genes that showed a 1.5-fold change in expression after antibiotic treatment when compared with the expression in control cells that were not exposed to antibiotics. In response to Amp, Km, Tc, and Nor, the expression levels of 1054 (26.6%), 1497 (37.33%), 1170 (29.52%), and 208 (5.25%) genes were markedly upregulated, and the levels of 1738 (43.86%), 910 (22.96%), 1254 (31.64%), and 635 (16.02%) genes were downregulated, respectively (Figure 1A, Table S1). The change in the expression of the same genes in response to each antibiotic treatment suggested that common responses were elicited by the 4 classes of antibiotics: 41 and 14 genes were commonly upregulated and downregulated, respectively (Figure 1B, Table 1, Table S2). Several upregulated genes appear to encode hypothetical proteins, a redox-sensing regulatory protein (*soxR*), RNA polymerase sigma factor (*rpoE*), dehydrogenases, and numerous transporter proteins. The commonly downregulated genes encoded a glycosyltransferase (*wcaA*), a lipoprotein (*rlpA*), and 3-dehydroquinate dehydratase (*aroQ*) (Table S2). Our RNA-Seq results were confirmed with quantitative real-time PCR (qRT-PCR). Commonly up- and down- regulated genes (*soxR, rpoE, lysR, wcaA*) and specifically induced genes were selected based on expression vales in 4 antibiotics conditions (Figure S2).

**Table 1:** Genes in *A. oleivorans* DR1 commonly upregulated by Amp, Km, Tc, and Nor.

Locus_tag DR1	Product	Genes	Fold-change			
			Amp	Km	Tc	Nor
AOLE_02445	Enoyl-CoA hydratase	*caiD*	4.62	1.78	2.34	1.87
AOLE_04025	Metal-dependent hydrolase		3.84	2.97	4.43	2.45
AOLE_06735	Putative short-chain dehydrogenase		4.15	2.37	3.63	1.92
AOLE_06795	Alkylhydroperoxidase		2.94	2.23	2.92	1.58
AOLE_08565	AraC-type DNA-binding domain-containing protein	*araC*	15.35	3.55	3.11	2.10
AOLE_08595	3-Oxoadipate enol-lactonase	*mhpC*	3.08	1.78	2.73	1.57
AOLE_08710	3-Oxoacyl-(acyl-carrier-protein) reductase	*fabG*	4.18	4.13	3.10	2.09
AOLE_08725	NIPSNAP family protein		3.35	3.55	6.22	2.09
AOLE_08765	Shikimate dehydrogenase	*aroE*	15.61	7.14	12.13	5.26
AOLE_09075	Transcriptional regulator	*lysR*	5.71	4.27	2.03	1.68
AOLE_09435	DoxX family protein		2.24	7.09	5.44	1.57
AOLE_09590	Putative tonB-like protein	*tonB*	1.57	3.90	3.42	1.67
AOLE_10175	Putative aliphatic sulfonate-binding protein	*tauA*	2.69	2.04	5.02	1.65
AOLE_11820	Major facilitator superfamily transporter	*araJ*	144.14	3.30	5.23	2.84
AOLE_11830	Methyltransferase domain-containing protein	*ubiE*	157.57	3.56	4.10	1.84
AOLE_12115	DMT-family permease		3.07	2.36	3.88	2.44
AOLE_12135	Redox-sensitive transcriptional activator SoxR	*soxR*	2.52	4.00	2.53	2.88
AOLE_12655	ECF subfamily protein RNA polymerase sigma-24 subunit	*rpoE*	5.05	3.56	1.82	1.75
AOLE_12705	Glycine betaine ABC transporter substrate-binding protein	*tauA*	1.71	1.77	1.75	2.49
AOLE_12875	Phenylacetic acid degradation protein	*paaI*	17.54	3.56	4.68	2.10
AOLE_13495	Competence-damaged family protein	*cinA*	5.05	13.36	7.42	2.63
AOLE_14540	Peptide deformylase	*def*	2.87	4.45	1.85	2.75
AOLE_14590	3-Phenylpropionate dioxygenase ferredoxin	*nirB*	2.44	2.85	3.75	1.58
AOLE_14800	RNA polymerase sigma factor FecI	*rpoE*	27.98	17.79	1.87	2.94
AOLE_16560	Short-chain dehydrogenase		1.71	2.44	2.24	1.57
AOLE_18975	GNAT family acetyltransferase		5.39	6.56	2.78	1.70

### Effects of antibiotics on the expression of specific genes

Clusters of orthologous groups (COGs) were analyzed to examine specific gene-expression changes (Figure S3). DR1 cells treated with Amp exhibited altered expression of several COG categories: translation (COG J), transcription (COG K), and inorganic-ion transport/metabolism (COG P) categories were mainly downregulated, whereas lipid metabolism (COG I) and amino-acid metabolism and transport (COG E) categories were upregulated. By contrast, Km and Tc treatments boosted the expression of gene clusters involved in transcription (COG K), amino-acid metabolism/transport (COG E), and inorganic-ion transport/metabolism (COG P) categories, whereas the treatments downregulated the expression of gene clusters involved in cell-wall/membrane/envelop biogenesis (COG M). Under Nor treatment, most COG categories were not changed to the same degree as they were changed in response to other antibiotics; certain genes involved in inorganic-ion transport/metabolism (COG P) were upregulated and transcription genes (COG K) were downregulated (Figure S3). COG analyses of the transcriptomes revealed that the genes associated with amino-acid metabolism and transport and inorganic-ion transport and metabolism are critical for cellular-stress and cell-death responses under all antibiotic-treatment conditions. Our data suggest that amino-acid metabolism and transporter systems might play key roles in antibiotic-resistance mechanisms in *Acinetobacter* species.

Distinct antibiotics possess specific cellular targets such as DNA, RNA polymerase, ribosomal proteins, and cell walls [18]. The overexpression of the antibiotic targets could enhance the survival of bacterial cells under antibiotic treatment [19]. Our data showed that specific antibiotic targets were strongly upregulated under distinct antibiotic conditions. The DR1 genome contains 9 putative lactamase genes. Interestingly, not all β-lactamases were upregulated by Amp, and class-C-type β-lactamases were primarily induced by Amp (Table 2). The expression of genes encoding penicillin-binding proteins was downregulated under Amp treatment (Table 2), which might be because of the high concentration of Amp used. Km treatment downregulated several ribosomal-protein genes (data not shown). However, Tc induced the expression of several ribosomal-protein genes, including the expression of genes encoding ribosomal proteins S13 and S7, which are recognized to interact directly with Tc [20]. Moreover, Tc treatment induced enzymes that modify ribosomal proteins, such as 50S ribosomal-protein methyltransferase and 30S ribosomal-protein methylthiotransferase. Thus, although Km and Tc inhibit translation by binding to ribosomes, their influences on cellular responses appeared to differ. Cells treated with Nor exhibited 2.92- and 2.8-fold increases in the expression of *gyrA* (AOLE_18380) and *gyrB* (AOLE_00595), respectively, which are recognized as targets of fluoroquinolone-class antibiotics (Table 2). Interestingly, Tc and Nor did not induce any lactamase genes, but Km induced class-C β-lactamase (AOLE_17635) and metallo-β-lactamases (AOLE_00775 and AOLE_03925). *Acinetobacter* species appear to express numerous efflux-pump genes that are critical for the multidrug resistance of *A. baumannii* [21]. However, based on our transcriptome data, it is unclear which efflux pumps are crucial for conferring resistance against the antibiotics tested (Table 2). Certain efflux pumps might be specific to each antibiotic.

**Table 2:** Antimicrobial resistance-associated genes and target genes in *A. oleivorans* DR1.

Locus_tag DR1	Product	Description	Genes	Fold-change			
				Amp	Km	Tc	Nor
**β-Lactamases**							
AOLE_05220	β-Lactamase	Class C β-lactamase	*ampC*	1.05	−2.06	−3.45	−1.31
AOLE_06930	β-Lactamase class C	Class C β-lactamase	*ampC*	5.20	1.93	1.03	1.00
AOLE_12585	β-Lactamase	Class C β-lactamase	*ampC*	1.41	1.19	1.72	−1.09
AOLE_17635	β-Lactamase class C	Class C β-lactamase	*ampC*	4.20	3.44	1.53	1.25
AOLE_11070	β-Lactamase class D	Class D β-lactamase	*bla_oxa-66_*	1.28	−2.14	−1.43	−1.65
AOLE_00775	Metallo-β-lactamase superfamily protein	Metallo-β-lactamase superfamily	*fpaA*	−2.31	2.51	1.51	−2.19
AOLE_03925	Putative metallo-β-lactamase	Metallo-β-lactamase superfamily	*gloB*	3.17	2.56	−2.55	−1.06
AOLE_10040	β-Lactamase	Metallo-β-lactamase superfamily	*gloB*	−1.32	1.72	−3.28	−1.11
AOLE_17515	Metallo-β-lactamase superfamily protein	Metallo-β-lactamase superfamily	*gloB*	−1.17	−1.05	−1.33	−1.05
AOLE_01440	Penicillin binding protein transpeptidase domain protein		*ftsI*	−1.03	−1.20	−1.62	−1.03
AOLE_01470	Putative penicillin-binding protein (PonA)		*mrcA*	−2.22	−4.35	−3.34	−1.11
AOLE_05610	Penicillin-binding protein 1B		*mrcB*	−1.80	−3.78	−3.30	−1.04
AOLE_14240	Penicillin-binding protein 2		*ftsI*	−4.09	−1.47	−1.68	−1.16
**Aminoglycosides**							
AOLE_08490	Predicted aminoglycoside phosphotransferase	aminoglycoside 6′-acetyltransferase	*aacA4*	3.93	1.30	−1.65	−1.07
AOLE_18475	Aminoglycoside2'-N-acetyltransferase(AAC(2′)-Ib)	aminoglycoside 2′-acetyltransferase	*aacB*	−8.13	2.15	1.99	−1.03
**Fluoroquinolones**							
AOLE_00020	DNA gyrase subunit B	GyrB mutation	*gyrB*	−1.82	−1.65	−1.48	1.24
AOLE_00595	DNA topoisomerase IV subunit B	GyrB mutation	*gyrB*	−1.18	1.61	−1.62	2.80
AOLE_04195	DNA gyrase subunit A	His-78→Asn	*gyrA*	−2.17	−1.51	−3.26	−1.01
AOLE_18380	DNA topoisomerase IV subunit A	GyrA mutation	*gyrA*	−1.22	−1.56	−2.00	2.92
**Efflux pumps**							
AOLE_00955	MFS transporter, metabolite:H+ symporter (MHS) family protein	MFS-family efflux pump	*uhpC*	−3.37	1.97	1.31	1.00
AOLE_00175	MFS-family transporter	MFS-family efflux pump	*araJ*	−1.63	4.02	2.99	1.38
AOLE_01040	MFS-family transporter	MFS-family efflux pump	*araJ*	−4.27	−3.45	1.14	−1.32
AOLE_12350	MFS-family transporter	MFS-family efflux pump	*araJ*	−4.03	−2.09	1.02	1.13
AOLE_00050	RND-type efflux pump	RND-family efflux pump	*dctP*	9.53	−1.03	1.52	1.02
AOLE_04230	Putative RND-family drug transporter	RND-family efflux pump	*emrA*	2.16	−15.53	−2.57	−1.21
AOLE_09410	RND-type efflux pump	RND-family efflux pump		6.59	1.19	3.25	1.58
AOLE_18750	RND-superfamily exporter	RND-family efflux pump		77.60	19.11	1.34	−1.13
AOLE_00035	ABC transporter ATP-binding protein	ABC-family efflux pump	*uup*	−3.32	−1.18	−2.10	1.22
AOLE_01345	Putative ABC transporter ATP-binding protein	ABC-family efflux pump	*uup*	−3.46	1.23	−3.34	1.06
AOLE_17260	ABC transporter ATP-binding protein	ABC-family efflux pump	*uup*	−3.46	−2.54	−3.51	−1.01
AOLE_00290	Multidrug-resistance protein norM	MATE-family efflux pump	*norM*	−3.58	−1.34	1.92	−1.03
AOLE_00530	Na+-driven multidrug efflux pump	MATE-family efflux pump	*norM*	2.40	1.14	1.73	1.16
AOLE_05880	MATE efflux family protein	MATE-family efflux pump	*norM*	-2.29	−1.69	2.25	1.28
AOLE_17460	Multidrug ABC transporter	MATE-family efflux pump	*norM*	−3.07	1.57	−1.22	−1.20
AOLE_05535	Quaternary ammonium compound-resistance protein QacE	SMR-family efflux pump	*qacE*	−2.23	2.48	1.71	1.47
AOLE_16200	Quaternary ammonium compound-resistance protein SugE	SMR-family efflux pump	*sugE*	−1.59	4.62	1.97	1.15

Interestingly, our data showed that the expression of fimbriae/pili-related genes changed in response to treatment with Amp, Km, and Tc (Table S3). Previously, we reported that *A. oleivorans* DR1 possesses 2 major fimbrial appendages [22]. Expression of fimbrial and pilin proteins is consistent with the cell aggregation and biofilm formations [23], and flagellar/pili appendages can function as transporting or adhering machines in gram-negative bacteria [24]. Other transcriptome data demonstrated that the flagellar/pili metabolism-related genes were induced under diverse stress conditions such as osmotic stress, oxidative stress [25], [26]. Beyond affecting target-gene expression, antibiotics are considered to exert secondary effects that are a part of the adaptive response to antibiotic stress.

### Effect of distinct classes of antibiotics on physiology: cell morphology and membrane permeability

**Figure 2:**
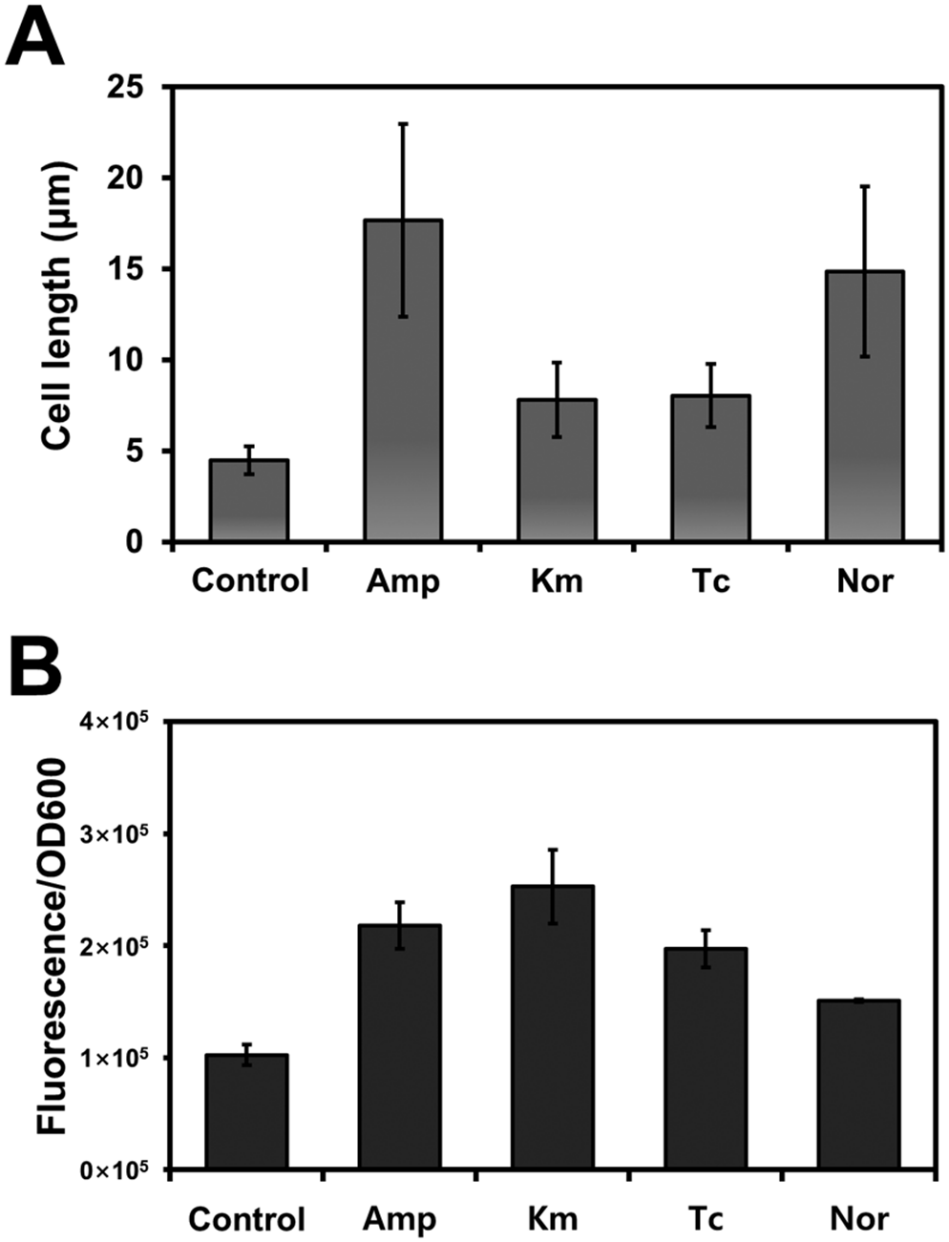
Influence of distinct classes of antibiotics on cell morphology, and membrane permeability in DR1. (A) The average cell size was measured from 50 cells treated with antibiotics. (B) Membrane permeability was measured using ANS. The error bars indicate standard deviation from triplicate experiments.

**Figure 3:**
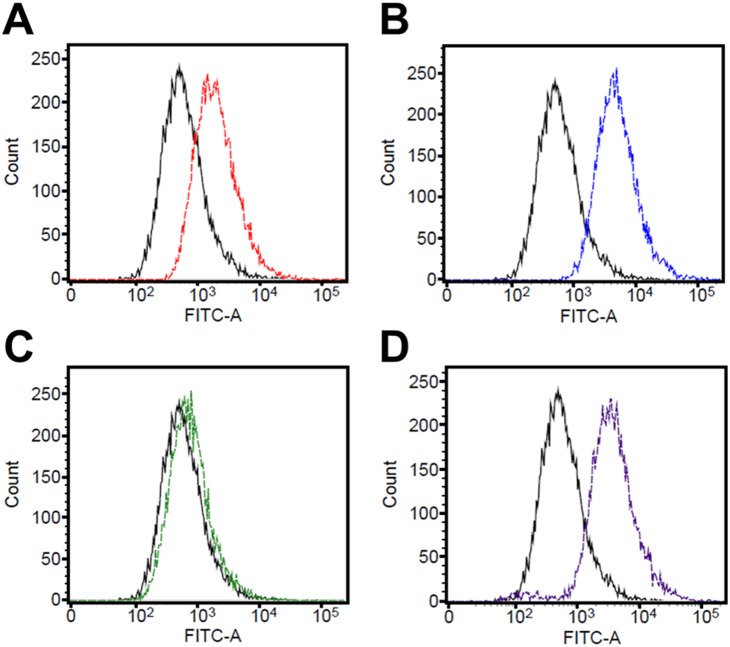
Measurement of oxidative stress induced by antibiotics. Intracellular superoxide-anion generation was measured using DHR 123. Fluorescence intensity was determined using flow cytometry and is represented as a histogram. FITC-A indicates the intensity of green fluorescence and the number of cells exhibiting the corresponding fluorescence intensity (amount of ROS production). The fluorescence histograms are of the samples before and after antibiotic treatment; solid and dotted lines are untreated cells and antibiotic-treated cells, respectively. (A) Amp, (B) Km, (C) Tc, (D) Nor. A shift to stronger fluorescence indicates a greater generation of oxidative stress.

We investigated the effects of antibiotics on physiological changes such as cell death and alterations in cell morphology and membrane permeability in an effort to link gene-expression to physiology. Examination of the morphology of cells stained with 4′,6-diamidino-2-phenylindole (DAPI) showed that cells treated with antibiotics were longer than wild-type cells (Figure 2A, Figure S4), and the cells treated with Amp and Nor were nearly 4-times longer than control cells (Figure 3A, B). Previously, β-lactams were reported to lengthen cells by inhibiting peptidoglycan biosynthesis [27]. Cell filamentation is often associated with the SOS response [28]. The product of the *sulA* gene, a key component of the SOS response that leads to cell elongation by binding to FtsZ or DpiAB in a two-component system, induces cell filamentation [29]. Interestingly, no *sulA* gene homolog is present in *Acinetobacter* species, and thus it is worth identifying the roles of other genes involved in cell elongation in *Acinetobacter* species. In the cell walls of most bacteria, peptidoglycans play an essential role in antimicrobial resistance; peptidoglycans determine cell shape, and their biosynthesis is critical for antibiotics resistance [30]. Peptidoglycan hydrolase is a widely conserved outer-membrane protein that modulates cell shape in *E. coli* and *Pseudomonas aeruginosa* [31]. The expression of AOLE_00215, which encodes peptidoglycan hydrolase, was increased 1.5- and 1.6-fold by Km and Tc, respectively, but was not markedly affected by Amp and Nor.

We measured the change in membrane permeability by using ANS, a neutral, hydrophobic fluorescent probe; in membrane-damaged cells, fluorescence is increased because the enhanced permeability leads to ANS uptake [32]. The fluorescence-intensity values measured were divided by the OD_600_ values for normalizing the measurements, and the results showed that distinct antibiotic treatments altered membrane permeability to different degrees (Figure 2B). The membrane-permeability properties have a major impact on the susceptibility of microorganisms to antibiotics [33]. Membrane permeability was increased substantially after Km treatment, whereas only a slight increase of membrane permeability was induced by Amp and Tc, which might explain the sensitive response of DR1 cells to Km. Porins are considered to be permanently open pores, and lowering porin expression reduces outer-membrane permeability [33]; thus, porin-mediated permeability is a critical aspect of antibiotic-resistance mechanisms. The DR1 genome contains several porin-encoding genes. The expression of *ompC* (AOLE_10405), which encodes an outer-membrane porin protein, increased 1.5-fold under Km treatment, but decreased in response to Amp (6.4-fold) and Nor (1.5-fold) and did not change after Tc treatment.

### Oxidative stress, SOS response, and DNA repair in response to distinct antibiotics

**Figure 4:**
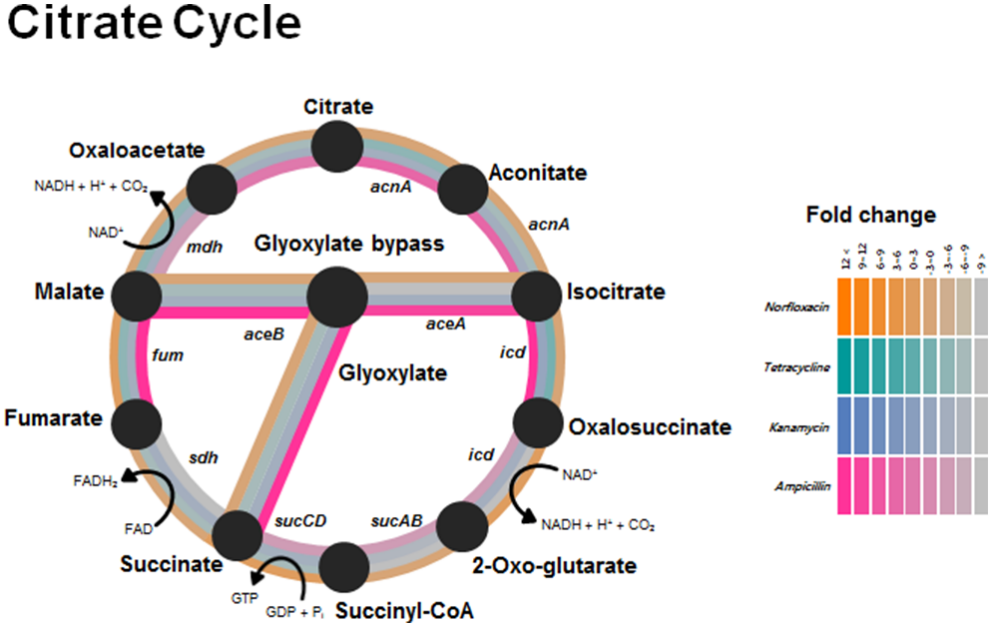
Expression of citrate-cycle genes in *A. oleivorans* DR1 treated with distinct antibiotics. Gene-expression changes are represented by a color gradient that is based on the fold-changes of gene expression in response to antibiotic treatments.

Antibiotics have been widely reported to induce the production of reactive oxygen species (ROS), which causes oxidative stress damage [34]. We used the fluorescent probe DHR 123 and flow cytometry to monitor ROS generation following treatment with the 4 antibiotics (Figure 3): under the tested conditions, treatment with Amp, Km, and Nor, but not Tc, potently induced ROS generation. Interestingly, the expression profiles of oxidative stress-related genes were distinct following treatment with these antibiotics of different classes, based on which we suggest that distinct mechanisms exist that are used by bacteria for coping with disparate types and levels of oxidative stress induced by various antibiotics (Table 2). Peroxiredoxin (*ahpC)* and catalase (*katE1*) genes were induced by Amp and the thioredoxin (*trxA*) gene was highly upregulated by Km and Tc, whereas the redox-sensing regulatory gene *soxR* was induced by all antibiotics. Antibiotic-induced oxidative stress upregulated glyoxylate-bypass genes [35]. The expression levels of isocitrate lyase (*aceA*) and malate synthase (*aceB*) genes, which are link to glyoxylate bypass, were increased substantially in response to Amp and Nor, but not Tc and Km (Figure 4). These results suggest that distinct classes of antibiotics elicit different responses to oxidative stress by dissimilarly affecting the expression of genes associated with ROS defense and glyoxylate bypass.

**Figure 5:**
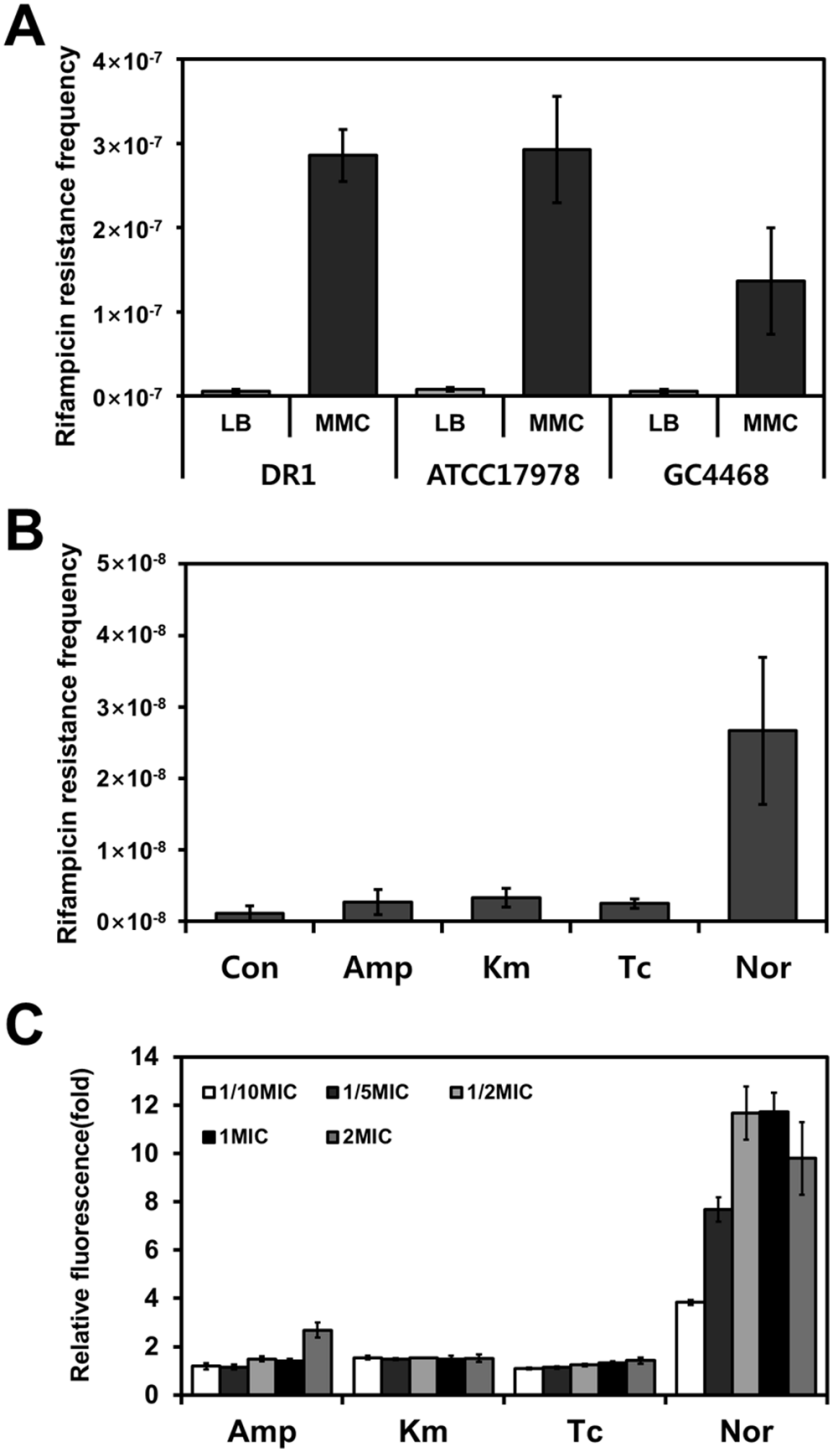
SOS-response induction in *Acinetobacter oleivorans* DR1. The mutation frequency, which corresponds to the rifampicin-resistance CFU count divided by the total CFU count, was measured and is represented on the Y-axis in the case of each antibiotic. (A) MMC-induced mutagenesis frequency. (B) Mutagenesis frequency induced by antibiotics of distinct classes. (C) Effect of antibiotics on *recA* expression was confirmed using a GFP fusion protein.

Unexpectedly, only Nor treatment substantially upregulated the expression of these SOS response-related genes and DNA-repair genes: *recA, umuDC, dinP, uvrAC*, and *ssb* (Table 3). The SOS response is a global response to DNA damage in bacteria that is induced by a variety of environmental factors such as UV radiation, chemicals, and antimicrobial compounds [36]. The RecA protein and LexA repressor play central roles in SOS response [37], [38], but a LexA-like transcriptional repressor has been studied only poorly in *Acinetobacter* species [39]. DNA damage increases the frequency of mutations when MMC is used, which indirectly confirms the presence of the SOS response [40]. Previously, MMC-induced mutation frequency was monitored by measuring the increase of colonies resistant to rifampicin [41]. MMC treatment increased the rifampicin-resistance mutation frequency 47-fold in DR1. When *E. coli* GC4468 and *A. baumannii* ATCC17978 were used as reference strains, the mutation frequency was determined to be increased 22- and 37-fold in *E. coli* and *A. baumannii*, respectively (Figure 5A). Our results reveal that crucial features of the canonical SOS response exist in the genome of DR1 cells. When we measured antibiotic-induced SOS response, we determined that rifampicin-resistance mutation frequency was strongly induced only by Nor (Figure 5B). Agreeing with these data, our reporter strains carrying GFP fused to the *recA* promoter region showed that Nor treatment induced the SOS response (Figure 5C). The fluorescence of these reporter cells depended on the concentration of Nor, although a high concentration of Amp increased *recA* expression. We could not rule out the possibility that *recA* transcription and GFP translation differ, because the RNA-seq results showed that *recA* expression increased under Km treatment. Antibiotic treatment can induce the SOS response, which can lead to the expression of *umuDC* [41]. Our transcriptome analysis revealed that the *umuDC* genes were induced only by Nor (Table 3). Thus, our results demonstrated that Nor, but not other antibiotics, strongly induced the SOS response in DR1 cells.

**Table 3:** Expression change of functional gene clusters.

Locus_tag DR1	Product	Genes	Fold change			
			Amp	Km	Tc	Nor
**SOS-response genes**						
AOLE_07085	Nucleotidyltransferase/DNA polymerase	*dinP*	−2.67	1.48	2.34	1.57
AOLE_07375	Recombinase A	*recA*	2.05	3.04	1.02	5.17
AOLE_07965	DNA-directed DNA polymerase UmuC	*umuC*	−1.43	−2.26	2.24	4.20
AOLE_07970	DNA polymerase V component		−1.43	−1.69	−1.10	5.41
AOLE_11745	SOS-response transcriptional repressor (RecA-mediated autopeptidases)	*umuD*	−1.18	1.82	−1.57	3.15
AOLE_14875	DNA polymerase V component		1.18	3.20	2.18	6.51
AOLE_14880	DNA-directed DNA polymerase UmuC	*umuC*	−4.42	−2.49	1.51	2.13
AOLE_18420	DNA polymerase IV	*dinP*	−1.67	1.34	−1.01	−1.53
**DNA repair-related genes**						
AOLE_05830	Putative DNA-binding/iron metalloprotein/AP endonuclease		−8.18	2.00	−1.26	−1.33
AOLE_13505	Metalloendopeptidase-like membrane protein	*nlpD*	2.96	1.35	−2.53	1.06
AOLE_14215	Endonuclease III	*nth*	−3.09	1.81	−1.31	−1.19
AOLE_14840	HNH endonuclease		−1.04	5.00	−1.12	−1.43
AOLE_18425	Endoribonuclease L−PSP family protein	*tdcF*	−1.31	−1.42	−7.04	1.11
AOLE_18840	Endoribonuclease L-PSP family protein	*tdcF*	5.89	−2.73	1.24	−1.16
AOLE_03065	Formamidopyrimidine-DNA glycosylase	*mutM*	−2.32	1.72	−1.24	−1.03
AOLE_10805	Uracil-DNA glycosylase	*ung*	−2.90	1.89	−1.41	−1.02
**Oxidative stress-related genes**						
AOLE_01750	Cu/Zn superoxide dismutase	*sodC*	2.99	−1.24	1.10	−1.07
AOLE_02915	Peroxiredoxin	*ahpC*	2.72	−1.38	−3.47	−1.98
AOLE_05305	Superoxide dismutase	*sodA*	−1.57	−1.27	−1.54	−1.16
AOLE_07635	Thioredoxin	*trxA*	−1.77	5.36	8.61	2.10
AOLE_11770	Catalase	*katE*	3.98	1.22	1.56	−1.17
AOLE_12135	Redox-sensitive transcriptional activator SoxR	*soxR*	2.52	4.00	2.53	2.88
AOLE_12755	Catalase	*katE*	1.41	1.54	2.09	−1.75
AOLE_13380	Peroxiredoxin	*ahpC*	1.23	1.24	−1.87	−1.23
AOLE_14380	Hydrogen peroxide-inducible genes activator	*oxyR*	1.11	1.40	−1.79	1.08
AOLE_16430	Thioredoxin	*trxA*	1.09	−1.24	−2.07	−1.09
AOLE_17390	Catalase	*katG*	−1.19	−3.11	−2.88	1.34
AOLE_18445	SoxR-family transcriptional regulator	*soxR*	1.17	1.38	1.47	−1.32

### Loss of DNA-repair capability in response to Km and Tc treatment

**Figure 6:**
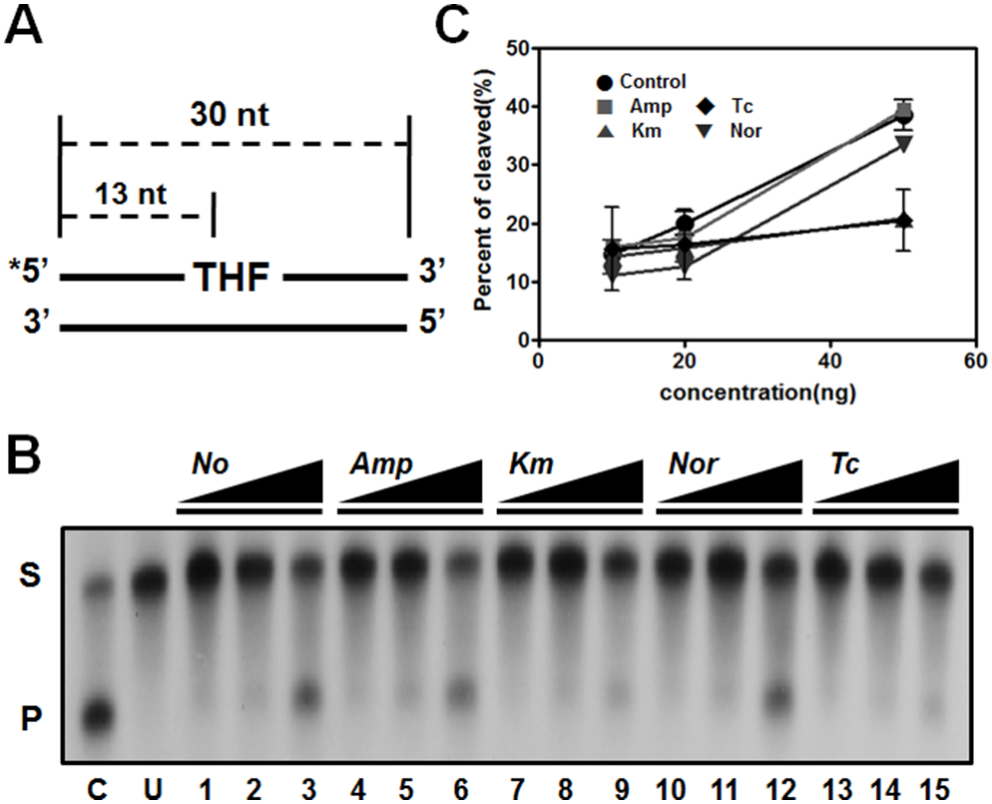
Verification of endonuclease IV activity by using the base-excision DNA-repair assay. DNA-repair capability of endonuclease IV was measured in DR1 exposed to distinct classes antibiotics. (A) Schematic representation of DNA substrate containing a site-specific THF residue. (B) A representative autoradiograph of gel electrophoresis to measure *in vitro* BER products. (C) Quantification of endonuclease IV BER activity. S, substrate; P, product; C, positive control; U, untreated negative control. Error bars indicate the S.D. calculated for each data point (n = 2).

The enzymes used in base excision repair (BER) are responsible for repairing endogenous DNA-damage lesions caused by ROS, environmental chemicals, and ionizing radiations [42], [43]. BER is a highly conserved cellular mechanism in bacteria and humans [42], and the lesion in the damaged DNA is removed by a DNA glycosylase. Endonuclease IV, UDG, and Fpg are induced in response to oxidative stress and these molecules function in repairing DNA damage in *E. coli* [44]. We measured endonuclease activity after treatment with the 4 antibiotics and we used the DNA-excision assay and oligonucleotides including THF residues [44]. Unexpectedly, in response to Km and Tc, endonuclease IV did not exhibit BER activity that was distinct from the activity in control (Figure 6). We also tested the activities of the 2 other DNA-repair enzymes, UDG and Fpg (Figure S5). Fpg activity decreased under all antibiotic conditions, whereas UDG activity was not changed. In these assays, enzyme reactions performed using purified *E. coli* endonuclease IV, UDG, and Fpg served as positive controls. Our results showed that the DNA-repair capability of endonuclease IV was maintained only under Amp and Nor treatment, which suggests that each antibiotic distinctly affects the genes encoding DNA-repair enzymes. The expression of endonuclease IV (AOLE_14840) was upregulated by Km but not the other 3 antibiotics, and the expression of Fpg (AOLE_03065) was decreased 2.3-fold and increased 1.7-fold in response to Amp and Km, respectively, but was unaffected by Tc and Nor. Our data reveal that the activity of DNA-repair enzymes was not correlated with the expression of the genes encoding these enzymes.

## Discussion

In this study, we conducted a comparative transcriptome analysis and examined the physiological changes in soil-borne *A. oleivorans* DR1 exposed to antibiotics of distinct classes. Although the antibiotic resistance of *A. baumannii* has been widely studied [45], the transcriptional response elicited by various antibiotics in other *Acinetobacter* species remains poorly documented. The effects of antibiotics and the antibiotic-resistance mechanism in DR1 have been described previously [22], [46], [47], but this is first study in which the transcriptional changes induced in DR1 cells by 4 antibiotics have comparatively analyzed. Our results revealed that the MIC of Amp exhibited extremely high ranges, which could be due to high number of lactamases encoded by the DR1 genome. Amp was hydrolyzed by various β-lactamases present in the periplasm before Amp can reach its targets [48]. Moreover, Amp induced the genes involved in glyoxylate bypass (Figure 4). Glyoxylate bypass is induced in numerous bacteria when carbon and energy sources are scarce or when oxidative stress is generated [49], [50]. Copper stress, which causes oxidative stress, induced glyoxylate bypass in *Pseudomonas* [51]. Glyoxylate bypass was particularly induced under Amp and Nor conditions (Figure 4). Km strongly induced oxidative stress and caused growth defects, but could not induce glyoxylate bypass. Therefore, we speculated that there are other factors that induce glyoxylate bypass in DR1 under antibiotic conditions.

In *E. coli*, sublethal concentrations of aminoglycosides increased the expression of several genes involved in heat-shock response, such as *htpG*, *ibpA*, *groES,* and *asrA* [52]. Aminoglycosides also induced the Lon protease in *P. aeruginosa* [53]. Our data showed that genes encoding chaperones and proteases (DnaK, AOLE_19360; GroEL, AOLE_03915; GroES, AOLE_03910) exhibit high RPKM values under Km treatment. These results suggest that chaperones and proteases might play a key role in mistranslation under Km condition in DR1 cells. Our data showed that endonucleases did not exhibit DNA-repair capabilities in DR1 cells treated with Km and Tc. Intriguingly, only ribosome-targeting antibiotics caused a loss of DNA-repair capability; this is probably because of the long protein-maturation times required for DNA-repair enzymes. Antibiotics can interfere with the metabolic pathways of bacteria, and this can cause structural alterations in the bacterial cell wall and surface appendages including flagella, fimbriae, and pili [54]. Bacteria employ extracellular structures such as pili and fimbriae in attachment and invasion, biofilm formation, cell motility, and transport across membranes [55]. Km and Tc have similar target regions, and they inhibit protein synthesis by binding to the 30S subunit of the ribosome [11], [13]. Our transcriptomic data showed that Km and Tc markedly induced fimbriae/pili-related genes. Interestingly, these antibiotics also upregulated the natural competence-associated type-IV pilus-assembly proteins encoded by AOLE_15230 (3.5-fold) and AOLE_17785 (3.69-fold).

Fluoroquinolones can induce the SOS response [56], key regulators of which are the proteins LexA and RecA [35], [36]. However, the lack of a LexA homolog indicates a critical role of other regulators for SOS response in *Acinetobacter* species [57]. Here, transcriptome analysis demonstrated that in DR1 cells, Nor strongly induced genes involved in the typical SOS response and DNA-repair genes. The relative amounts of SOS gene expression are determined primarily through by transcriptional regulation. Our previous study showed that, Nor treatment caused target-gene mutation in *gyrA* (AOLE_04195) and persister formation in DR1 [46]. Our data additionally validated the SOS response of *Acinetobacter* species by showing that DNA damage enhanced mutation frequency. This characteristic of DR1 might be helpful for having resistance to antibiotics stress.

Noncoding RNAs are commonly referred to as small RNAs because they are 50–500 nucleotides in size [58]. Small RNAs are potent regulatory molecules that function at the transcriptional or posttranscriptional level [59]. Interestingly, RNA-seq mapping data revealed that the noncoding regions of DR1 contain sequences of small-RNA candidates (Table S4). Three small-RNA candidates are conserved in certain *Acinetobacter* species. The Northern blot analysis confirmed the expression of small-RNA candidates (Figure S6).

In a recent study on *A. baumannii*, 31 putative small RNAs were identified using computational approaches [60]. Two of these small RNAs display sequence similarities with those of the DR1 strain and other *Acinetobacter* species. However, these 2 small RNAs were not induced under our tested conditions. Small RNAs play key roles in efflux-pump regulation and antimicrobial-agent resistance in *A. baumannii* [60], and efflux pumps are widely accepted to bestow clinically relevant resistance to antibiotics [61]. How small RNAs involved in expression of efflux pumps remains to be investigated in DR1 cells. Our study will serve as a baseline for understanding the effects of antibiotics on *Acinetobacter* species, and it should help in developing a new strategy for predicting novel antibiotic-resistance mechanisms, as well as for preventing multidrug resistance across multiple species of bacteria by using this soil-borne bacterium.

## Materials and Methods

### Bacterial strains, growth conditions, and antibiotics

The bacterial strains used in this study are listed in Table S5. Environment-originated nonpathogenic *A. oleivorans* DR1 was grown in nutrient broth at 30°C with rotational shaking at 220 rpm. Bacteria harboring plasmids and wild-type bacteria were cultured under the same conditions. *Escherichia coli* GC 4468 and *A. baumannii* ATCC17978 were grown at 37°C in LB and aerated by means of shaking. In bacterial antibiotic-treatment experiments, we used commercially available Rifampicin (Sigma-Aldrich, USA), Amp (Bioshop, Canada), Km (Bioshop, Canada), Tc (Sigma-Aldrich, USA), and Nor (Sigma-Aldrich, USA).

### Determination of antibiotic minimum inhibitory concentrations (MICs) of *A*. oleivorans DR1

MICs were determined in liquid nutrient medium by using 96-well polystyrene microtiter plates (Costar, USA). DR1 cells were grown overnight in nutrient broth at 30°C with shaking at 220 rpm. The cells were washed twice with phosphate-buffered saline (PBS) and inoculated at a cell density of 10^5^∼10^8^CFU/mL in 200 µL of nutrient broth containing 0–256 µg/mL of each antibiotic (Amp, Km, Tc, Nor), and then grown in 96-well polystyrene plates at 30°C for 24 h without shaking. MICs were determined by measuring the optical density at 600 nm (OD_600_) by using a microtiter-plate reader (PowerWaveXS, Bio-Tek, USA); the MICs were the lowest concentrations of the 4 antibiotics at which OD_600_ was <0.04.

### RNA extraction, sequencing, and analysis

Total RNA of DR1 cells grown in nutrient media was isolated from exponential-phase cells (OD_600_∼0.4). Cells were grown at 30°C with shaking at 220 rpm and when they reached the exponential phase, they were treated without or with each antibiotic at the sub-MIC (Amp,100 g/mL, Km, 4 g/mL, Tc: 1 g/mL, Nor: 4 g/mL) for 15 min. Total RNA was extracted using RNeasy Mini kits (Qiagen, USA) by following the manufacturer’s instructions. The isolated RNA was stored at −80°C until use. All RNA-sequencing and alignment procedures were conducted by Chunlab (Seoul, South Korea). The RNA was subjected to a subtractive Hyb-based rRNA-removal process by using the MICROBExpress Bacterial mRNA Enrichment Kit (Ambion, USA), and subsequent processes, including library construction, were performed as described previously (Table S1) [62]. RNA sequencing was performed using 2 runs of the Illumina HiSeq to generate single-ended 100-bp reads. The genome sequence of *A. oleivorans* DR1 was retrieved from the NCBI database (accession number NC_014259.1). Quality-filtered reads were aligned to the reference-genome sequence by using the CLC Genomics Workbench 6.5.1 tool (CLC bio, Denmark). Mapping was based on a minimal length of 100 bp, with an allowance of up to 2 mismatches. The relative transcript abundance was measured in reads per kilobase of exon sequence per million mapped sequence rea20kds (RPKM) [63]. The mapping results were visualized using the CLRNAseq program (Chunlab, South Korea). The RNA-seq data were deposited in the National Center for Biotechnology Information (NCBI) GEO site under accession numbers GSE38340, GSE44428, GSE58166 and GSE58167.

### Quantitative real-time PCR (qRT-PCR) analysis

cDNA was synthesized from 1 µg each RNA extract by using gene specific primers. (Table S5) and the primers for genes were used as templates for quantitative real-time PCR (qRT-PCR). The 25 µl PCR mixture included 12.5 µl iQ SYBR Green Supermix (Bio-Rad, USA), 1 µl of each primer (0.5 µM), 2 µl cDNA, and 8.5 µl distilled water. The PCR reactions were conducted at 95°C for 3 min, followed by 40 cycles consisting of 30 s at 95°C, 30 s at 60°C, and 30 s at 72°C. The expression level of each gene was normalized to the 16S rRNA expression level that was quantified with 16s rRNA-341F/16s rRNA-534R primers. Relative quantifications were performed in triplicate.

### Cell membrane permeability assays

The fluorescent probe 8-anilino-1-naphthylenesulfonic acid (ANS; Sigma-Aldrich, USA) was used for assessing the integrity of bacterial cell membranes. Overnight cultures were diluted 100-fold in 5 mL of fresh medium and grown to the logarithmic-growth phase at 30°C and 220 rpm. After the cells were treated with or without each antibiotic at the exponential phase (OD_600_ ∼0.4) for 15 min, 1 mL of the cell cultures was harvested by centrifugation (13,000× *g*, 1 min) and washed twice with PBS. The resuspended solutions were supplemented with ANS (1 µL, 3 mM) and maintained at room temperature for 10 min in the dark. The fluorescence intensity of cells was measured using a microplate reader. The filter set used for fluorescence measurements included a 555-nm excitation filter and 590-nm emission filter. The possibility that distinct growth rates were measured under various experimental conditions was excluded by normalizing protein amounts (in µg). Cell membrane permeability assays were performed 3 times independently.

### Microscopic observation

Antibiotics (used at the sub-MICs) was added to the cells at the exponential phase (OD600nm = 0.4), and the cells were then incubated for 30 min at 30°C. 1 mL of the cell cultures was harvested by centrifugation (13,000× *g*, 1 min) and washed twice with PBS. The resuspended solutions were supplemented with 4′,6-diamidino- 2-phenylindole (DAPI) (1 µL, 2 µg/mL), and maintained at room temperature for 10 min in the dark. DAPI -treated cells was washed and resuspended using PBS. Then, 5 µL of cells was placed on a glass slide and observed. Bacteria treated with antibiotics were viewed with a Carl ZeissAxio Imager microscope (ZEISS, Germany).

### Measurement of oxidative stress

Intracellular superoxide-anion generation was measured using dihydrorhodamine (DHR) 123 (Sigma-Aldrich, USA). The cells were grown to exponential phase (OD_600_∼0.4) and treated for 15 min with the antibiotics (used at the sub-MICs). The cells were washed twice and resuspended using PBS and then treated with DHR 123 (2.5 µg/mL) for 1 h in the dark at 30°C. DHR-123-treated cells were washed and resuspended using PBS, and the intracellular superoxide anion-mediated oxidation of DHR 123 was assayed be means of FACSverse flow cytometry (BD Biosciences, San Jose, CA, USA). The samples were analyzed by using a fluorescein isothiocyanate (FITC) argon-ion laser for excitation, and fluorescence intensity was determined and analyzed by measuring 10,000 cell counts. BD FACSuite software was used for data analysis.

### DNA damage-induced mutagenesis frequency

Cells were grown to the exponential phase (OD_600_∼0.4) and treated without (control) or with 1 g/mL MMC (sub-MIC) and antibiotics (used at the sub-MICs) for 1 h. After the treatment, cells were washed twice and resuspended using PBS and inoculated at a cell density of 5×10^8^ CFU/mL in 5 mL of fresh nutrient broth and grown with shaking at the appropriate temperature for 24 h. The cultures were collected and diluted in PBS and then plated on nutrient or LB agar media containing either 100 µg/mL rifampicin or no rifampicin to calculate the frequencies of rifampicin-resistance mutations. Colonies were counted after incubation for 24 h at the appropriate temperatures. Mutation frequency was determined from the relative percentage of CFU/mL ((CFU at 100 g/mL rifampicin/CFU at no rifampicin) × 100).

### Construction of transcriptional-fusion green fluorescent protein (GFP) and quantification of GFP fluorescence

The broad-host-range expression vector pRK415 was used for constructing transcriptional-fusion GFP. A fragment of the *recA* promoter region was amplified by means of Polymerase chain reaction (PCR) performed using pRKprecA-gfp-F/pRKprecA-gfp-R primer pairs (Table S5). A 190-bp fragment of the promoter region of *recA* was cloned into the *Kpn*I/*Bam*HI cloning site of the multi cloning site of the pRK415 vector. The amplicon (715 bp) obtained using pRKgfp-F/pRKgfp-R was cloned into the *Bam*HI/*Eco*RI cloning site of the pRK415 vector to generate transcriptional-fusion GFP. The plasmid was extracted using a Dyne Plasmid Miniprep Kit (DYNEBIO, Korea). The constructed plasmid was then introduced into *E. coli* Top10 and A. *oleivorans* DR1 by electroporation. Competent cells (50 µl) were transformed with 2.5 µl plasmid DNA samples using a Micropulser (Bio-Rad, USA) with a time constant range of 3.0–3.5 ms and a constant voltage of 4.5–5 kV. PCR was conducted to confirm insertion of the gfp gene using the GFP-F/GFP-R primer set (Table S5). Overnight cultures of the DR1 harboring constructing transcriptional-fusion GFP grown in nutrient broth were diluted 100-fold in 5 mL of fresh medium and then incubated with shaking. At the exponential-growth phase (OD_600_∼0.4), the antibiotics were added and the cells were incubated for 1 h. A 1-mL aliquot of each GFP fusion-strain culture was harvested and centrifuged at 13,000×*g* for 1 min and then washed twice with PBS. The resuspended cells were transferred to polystyrene 48-well microtiter plates (BD Biosciences, USA) and the GFP fluorescence intensity of the cells was quantified using a Multi-Detection Microplate Reader (Sense, HIDEX, Finland). The GFP fusion-strain expressed a stable GFP variant that has an excitation wavelength of 488 nm and emission wavelengths of 507–510 nm. The OD_600_ of each culture was measured using a microtiter-plate reader (PowerWaveXS; Bio-Tek, USA). The possibility of distinct growth rates being measured under various experimental conditions was excluded by normalizing the measured fluorescence intensity relative to the OD_600_ value. One fluorescence unit was defined as [(fluorescence intensity of cells/fluorescence intensity of PBS buffer)/OD_600_ of cells], and a relative fluorescence unit (fold) was defined as [fluorescence unit of treated cells/fluorescence unit of control (untreated) cells].

### *In vitro* base-excision repair (BER) assay

Overnight cultures were diluted 100-fold in 500 mL of fresh nutrient medium and grown to the exponential phase (OD_600_∼0.4) at 30°C and 180 rpm. After the cells were treated with or without antibiotics for 15 min, a cell cultures was harvested by centrifugation (10,000×*g*, 30 min) and washed twice with PBS. The cell pellet was resuspended in <5 ml of sonication buffer (50 mM Tris-HCl (pH 8.0), 1 mM EDTA, and 0.1 mM DTT), and cells were lysed by sonification. After cell debris was removed by centrifugation (13,000×*g*, 20 min) at 4°C, supernatant was collected and placed on ice. Cell-free extract was transferred to Eppendorf tubes (0.5 mL) and stored in 100 µl aliquots at −80°C. The radionucleotide [γ-^32^P] ATP was obtained from PerkinElmer Life Sciences (Wellesley, USA). We purchased *E. coli* uracil-DNA glycosylase (UDG), formamidopyrimidine-DNA glycosylase (Fpg), endonuclease IV, and T4 polynucleotide kinase (New England Biolabs, UK). Micro Bio-Spin 30 Chromatography Columns were from Bio-Rad. DNA oligonucleotides containing uracil, tetrahydrofuran (THF), or 8*-*oxoguanine (8-oxoG) residues were provided by Dr. B. Demple, SUNY*-*Stony Brook (Stony Brook, USA), and these were amplified by means of PCR performed using 30F-F/30F-R, U30-F/U30-R, OxoG-F/OxoG-R primer pairs (Table S5). The endonuclease-IV-activity assay was performed in a reaction mixture 10 µL containing 50 mM Hepes-KOH (pH 7.5), 8 mM MgCl_2_, 5% glycerol, 0.5 mM DTT, 0.1 mg/mL BSA, and 1 nM 5′-end-labeled duplex-DNA substrate containing THF residues. The reactions were initiated by adding 10, 20, or 50 ng of cell-free extracts and were incubated at 37°C. Aliquots of each reaction were withdrawn at 30 min, and the reactions were terminated by adding formamide loading buffer. The reaction products were separated by performing electrophoresis; we used 15% denaturing polyacrylamide gels containing 7 M urea in 90 mM Tris, 90 mM boric acid, and 2 mM EDTA. Gels were dried using a gel dryer (Model 583, Bio-Rad, USA), and products were visualized by means of autoradiography and quantified using ImageQuant software v5.2. The percentage of cleaved AP sites was calculated from amount of products divided by the sum of total products and substrates.

### Northern blot assay

Total RNA (5 µg) were run on denaturing agarose gels containing 0.25 M formaldehyde, and the gels were stained with ethidium bromide (EtBr) to visualize 23S and 16S rRNA. The fractionated RNA was transferred to nylon membranes (Schleicher & Schuell, Germany) using a Turboblotter (Schleicher & Schuell, Germany). The mRNA levels were determined by hybridizing the membrane with a gene specific, ^32^P-labeled probe (Takara, Japan) prepared by PCR amplification with their respective primer pair as indicated in Table S5. Autoradiography was conducted using an IP plate (Fujifilm, Japan) and a Multiplex Bio-Imaging system (FLA-7000; Fujifilm, Japan).

## Supporting Information

Figure S1 **Determination of MIC under different cell density in** ***A*** **. oleivorans DR1.**
(TIF)

Figure S2 **Confirmation of RNA-Seq results with qRT-PCR.** (A) Commonly up- and down- regulated genes were confirmed the gene expression on 4 antibiotics conditions. (B) Three genes were selected based on expression value on each antibiotics condition.
(TIF)

Figure S3 **COG assignments of differently expressed genes under distinct antibiotics conditions.** The percentage of up-regulated and down-regulated genes was sorted by general COG categories. Colors of the bars indicate the changes of gene expression. Red, gene expression is >1.5-fold change in RPKM value, Brown, gene expression is <1.5-fold change in RPKM value, Gray, gene expression of between a −1.5 and 1.5-fold change in value. COG abbreviations for the functional categories: A, RNA processing and modification; B, chromatin structure and dynamics; C, energy production and conversion; D, cell cycle control and mitosis; E, amino acid metabolism and transport; F, nucleotide metabolism and transport; G, carbohydrate metabolism and transport; H, coenzyme metabolism; I, lipid metabolism; J, translation, including ribosome structure and biogenesis; K, transcription; L, replication, recombination, and repair; M, cell wall structure and biogenesis and outer membrane; N, secretion, motility and chemotaxis; O, molecular chaperones and related functions; P, inorganic ion transport and metabolism; Q, secondary metabolite biosynthesis, transport, and catabolism; T, signal transduction; U, intracellular trafficking, secretion, and vesicular transport; V, defense mechanisms.
(TIF)

Figure S4 **Microscopic observation of antibiotics treated and untreated DR1 cells.** Morphology observation of cell treated with antibiotics. Phage contrast and staining with DAPI are shown. The scale bar represents 20 µm.
(TIF)

Figure S5 **Verification of UDG and Fpg activity by using the base-excision DNA-repair assay.**
(TIF)

Figure S6 **Confirmation of expression of small RNA candidates using Northern blot.** The expression of small RNA candidate was determined under antibiotics conditions using Northern blot. The ethidium bromide (EtBr) staining demonstrated consistent loading in all lanes.
(TIF)

Table S1 **Total number of reads aligning with the regions of interest (coverage) of the five libraries constructed from the RNA samples.**
(DOCX)

Table S2 **Fimbriae/pili related gene expression profiles by different class antibiotics.**
(DOCX)

Table S3 **Bacterial strains, plasmids, and primers used in this study.**
(DOCX)

Table S4 **The feature of small RNA genes in** ***A. oleivorans*** **DR1.**
(DOCX)

Table S5 **Bacterial strains, plasmid and oligonucleotides sequence used in this study.**
(DOCX)
