# Supplementary figures and images for: Global Transcriptome and Physiological Responses of Acinetobacter oleivorans DR1 Exposed to Distinct Classes of Antibiotics
Source: PLoS One. 2014 Oct 17;9(10):e110215. doi: 10.1371/journal.pone.0110215 (PMC4201530; doi:10.1371/journal.pone.0110215)

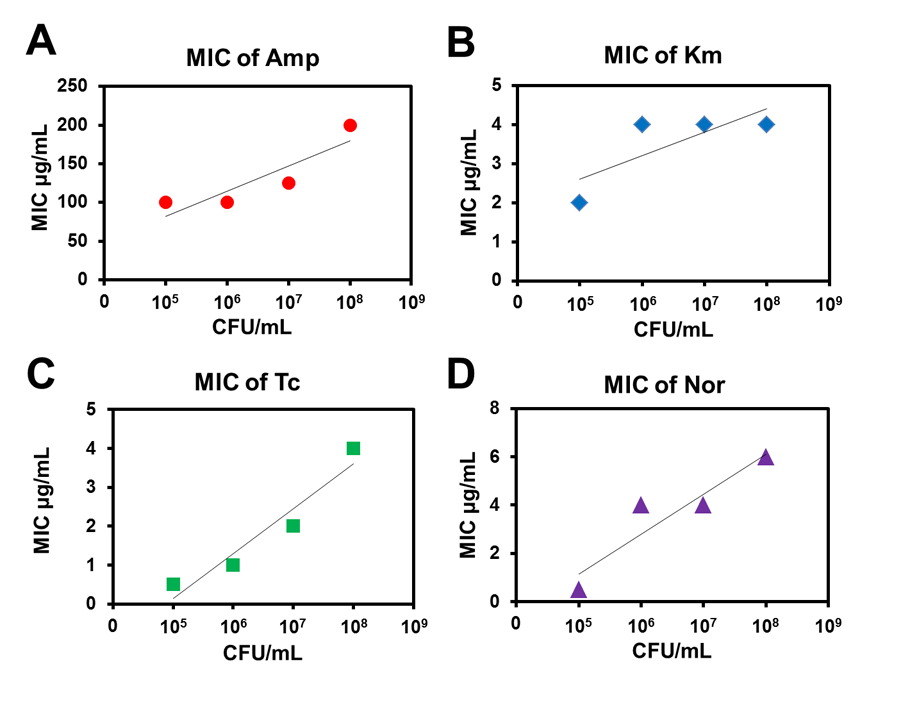

Supplement: Figure S1 — Determination of MIC under different cell density in A . oleivorans DR1. (TIF) [file pone.0110215.s001.tif]

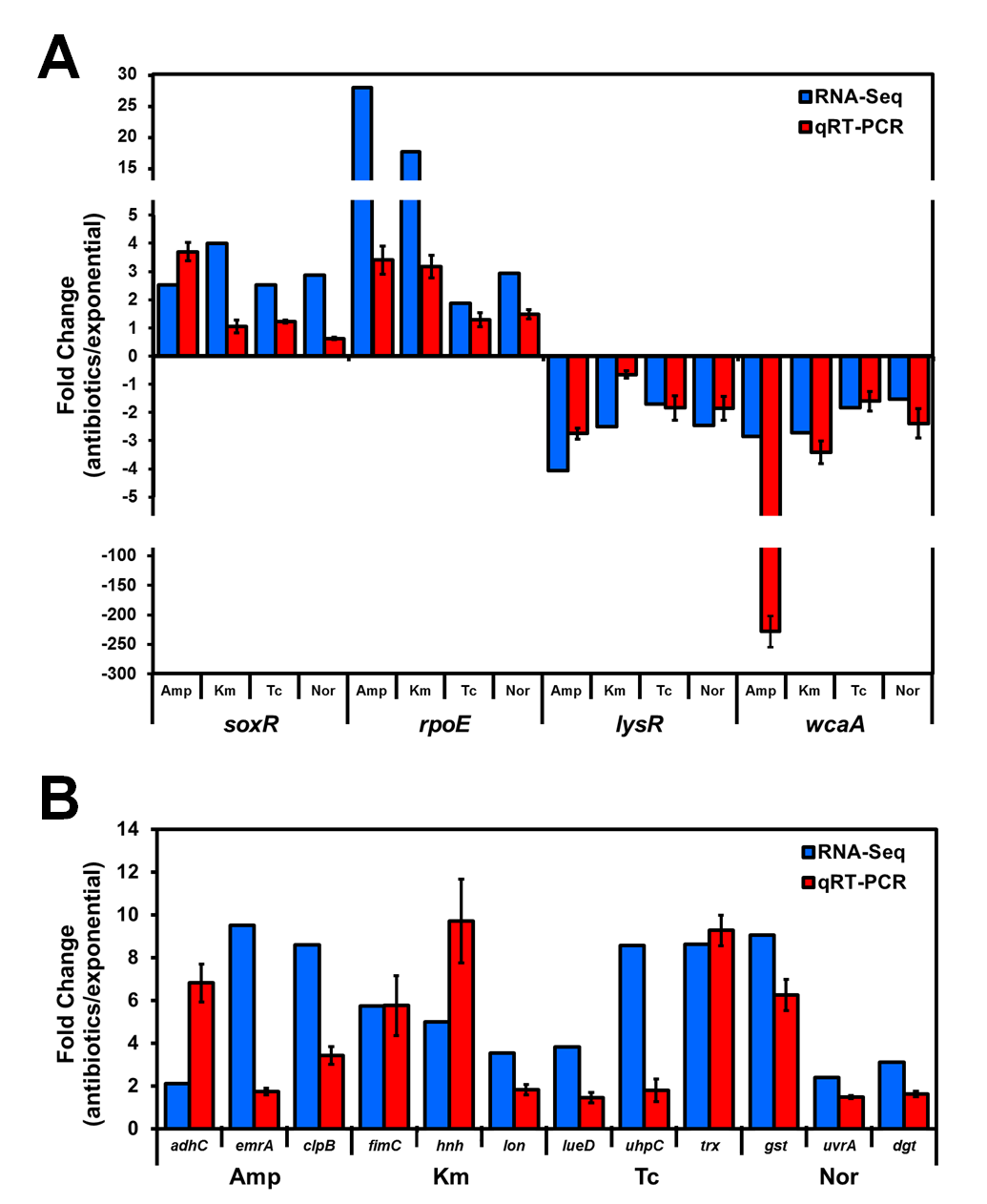

Supplement: Figure S2 — Confirmation of RNA-Seq results with qRT-PCR. (A) Commonly up- and down- regulated genes were confirmed the gene expression on 4 antibiotics conditions. (B) Three genes were selected based on expression value on each antibiotics condition. (TIF) [file pone.0110215.s002.tif]

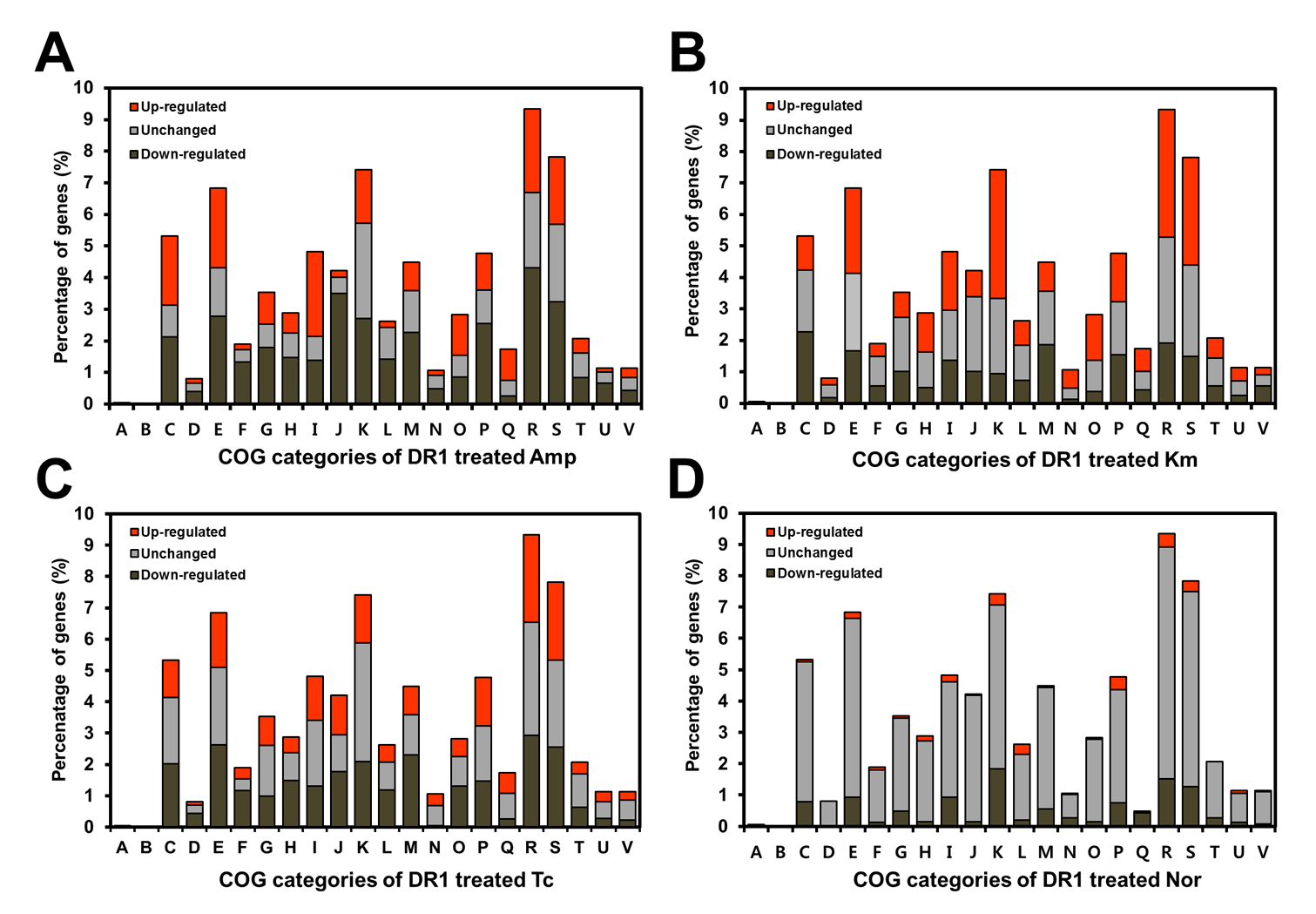

Supplement: Figure S3 — COG assignments of differently expressed genes under distinct antibiotics conditions. The percentage of up-regulated and down-regulated genes was sorted by general COG categories. Colors of the bars indicate the changes of gene expression. Red, gene expression is >1.5-fold change in RPKM value, Brown, gene expression is <1.5-fold change in RPKM value, Gray, gene expression of between a −1.5 and 1.5-fold change in value. COG abbreviations for the functional categories: A, RNA processing and modification; B, chromatin structure and dynamics; C, energy production and conversion; D, cell cycle control and mitosis; E, amino acid metabolism and transport; F, nucleotide metabolism and transport; G, carbohydrate metabolism and transport; H, coenzyme metabolism; I, lipid metabolism; J, translation, including ribosome structure and biogenesis; K, transcription; L, replication, recombination, and repair; M, cell wall structure and biogenesis and outer membrane; N, secretion, motility and chemotaxis; O, molecular chaperones and related functions; P, inorganic ion transport and metabolism; Q, secondary metabolite biosynthesis, transport, and catabolism; T, signal transduction; U, intracellular trafficking, secretion, and vesicular transport; V, defense mechanisms. (TIF) [file pone.0110215.s003.tif]

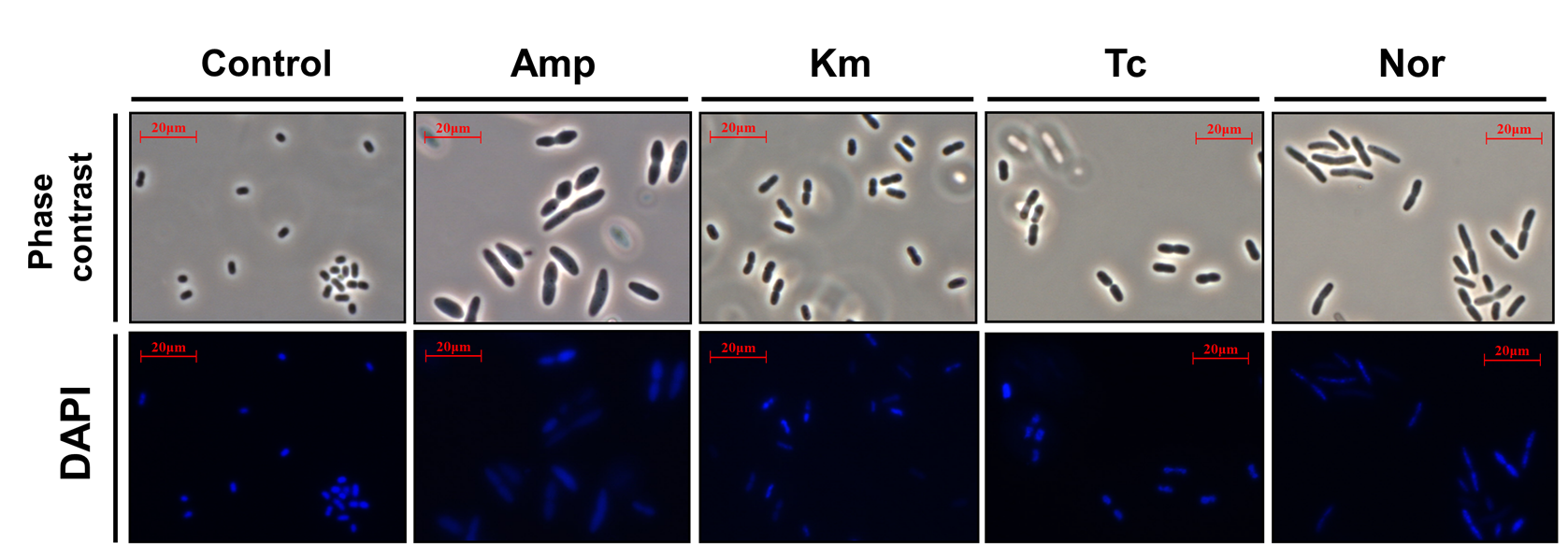

Supplement: Figure S4 — Microscopic observation of antibiotics treated and untreated DR1 cells. Morphology observation of cell treated with antibiotics. Phage contrast and staining with DAPI are shown. The scale bar represents 20 µm. (TIF) [file pone.0110215.s004.tif]

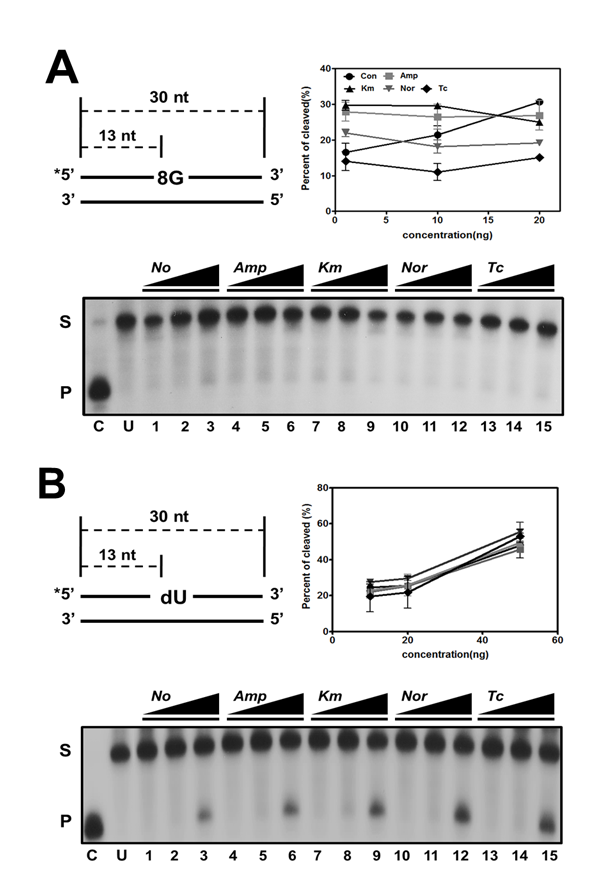

Supplement: Figure S5 — Verification of UDG and Fpg activity by using the base-excision DNA-repair assay. (TIF) [file pone.0110215.s005.tif]

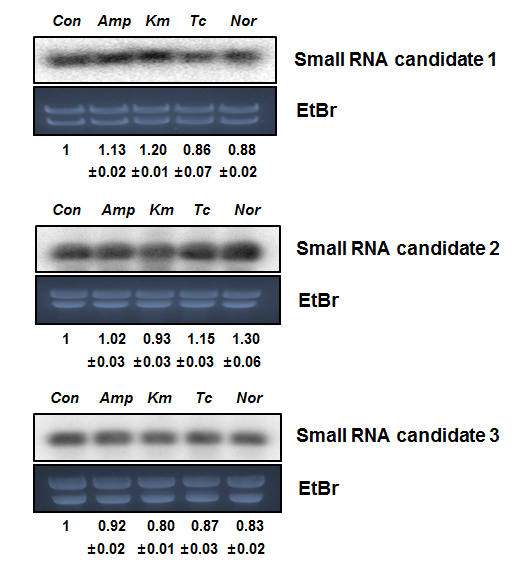

Supplement: Figure S6 — Confirmation of expression of small RNA candidates using Northern blot. The expression of small RNA candidate was determined under antibiotics conditions using Northern blot. The ethidium bromide (EtBr) staining demonstrated consistent loading in all lanes. (TIF) [file pone.0110215.s006.tif]
