# Supplementary material for: Global Transcriptome and Physiological Responses of Acinetobacter oleivorans DR1 Exposed to Distinct Classes of Antibiotics
Source: PLoS One. 2014 Oct 17;9(10):e110215. doi: 10.1371/journal.pone.0110215 (PMC4201530; doi:10.1371/journal.pone.0110215)
Supplement: Table S1 — Total number of reads aligning with the regions of interest (coverage) of the five libraries constructed from the RNA samples. (DOCX) [file pone.0110215.s007.docx]

**Table S1. Total number of reads aligning with the regions of interest (coverage) of the five libraries constructed from the RNA samples.**

|  | Total read | rRNA read | mRNA read | Intergenic read |
| --- | --- | --- | --- | --- |
| Exponential  (control) | 6583302 | 2387494 | 3536275 | 653515 |
| Amp | 38281074 | 805045 | 26922132 | 6300938 |
| Km | 52197392 | 25018598 | 4461169 | 11006119 |
| Tc | 17509940 | 11438553 | 4539284 | 1444725 |
| Nor | 19081119 | 14702179 | 337254 | 903790 |
