# Supplementary material for: Global Transcriptome and Physiological Responses of Acinetobacter oleivorans DR1 Exposed to Distinct Classes of Antibiotics
Source: PLoS One. 2014 Oct 17;9(10):e110215. doi: 10.1371/journal.pone.0110215 (PMC4201530; doi:10.1371/journal.pone.0110215)
Supplement: Table S2 — Fimbriae/pili related gene expression profiles by different class antibiotics. (DOCX) [file pone.0110215.s008.docx]

**Table S2. Commonly down-regulated genes in *A. oleivorans* DR1 by Amp, Km, Tc and Nor.**

| Locus_tag  DR1 | Product | Genes | Fold change | | | |
| --- | --- | --- | --- | --- | --- | --- |
|  |  |  | **Amp** | **Km** | **Tc** | **Nor** |
| AOLE_00825 | 16S ribosomal RNA methyltransferase RsmE | *rsmE* | -4.38 | -5.06 | -1.73 | -1.58 |
| AOLE_02335 | hypothetical protein | *tonB* | -3.62 | -1.92 | -1.64 | -1.68 |
| AOLE_02555 | hypothetical protein |  | -3.67 | -1.80 | -1.69 | -1.60 |
| AOLE_02560 | hypothetical protein |  | -5.74 | -2.33 | -1.96 | -1.84 |
| AOLE_02735 | hypothetical protein |  | -1.54 | -1.78 | -2.00 | -1.62 |
| AOLE_05440 | rare lipoprotein A | *rlpA* | -6.76 | -5.43 | -9.41 | -1.94 |
| AOLE_06200 | Binding-protein-dependent transport system inner membrane component family Protein | *abcD* | -10.18 | -6.90 | -4.31 | -1.59 |
| AOLE_07080 | 3-dehydroquinate dehydratase | *aroQ* | -9.05 | -1.75 | -5.27 | -1.83 |
| AOLE_07755 | hypothetical protein |  | -2.62 | -2.06 | -1.77 | -2.63 |
| AOLE_09650 | ABC transporter permease | *tauC* | -31.89 | -10.10 | -1.78 | -2.15 |
| AOLE_10515 | haloacid dehalogenase-like family hydrolase |  | -4.97 | -4.57 | -1.74 | -1.75 |
| AOLE_10630 | hypothetical protein |  | -1.67 | -1.53 | -2.75 | -1.68 |
| AOLE_11145 | LysR family transcriptional regulator | *lysR* | -4.07 | -2.51 | -1.70 | -2.46 |
| AOLE_16860 | glycosyltransferase | *wcaA* | -2.84 | -2.71 | -1.84 | -1.53 |
