# Supplementary material for: Global Transcriptome and Physiological Responses of Acinetobacter oleivorans DR1 Exposed to Distinct Classes of Antibiotics
Source: PLoS One. 2014 Oct 17;9(10):e110215. doi: 10.1371/journal.pone.0110215 (PMC4201530; doi:10.1371/journal.pone.0110215)
Supplement: Table S3 — Bacterial strains, plasmids, and primers used in this study. (DOCX) [file pone.0110215.s009.docx]

| Locus tag  DR1 | Product | Gene | Fold change | | | |
| --- | --- | --- | --- | --- | --- | --- |
|  |  |  | **Amp** | **Km** | **Tc** | **Nor** |
| AOLE_01545 | Tfp pilus assembly protein, major pilin PilA | PilA | 26.04 | 68.19 | 4.67 | -1.17 |
| AOLE_01600 | Tfp pilus assembly protein FimT | FimT | 36.27 | 4.89 | 2.14 | 1.05 |
| AOLE_01605 | Tfp pilus assembly protein PilV | PilV | 29.10 | 3.20 | 2.26 | 1.78 |
| AOLE_01610 | Tfp pilus assembly protein PilW | PilW | 14.17 | 1.73 | 1.11 | 0.51 |
| AOLE_01620 | Tfp pilus assembly protein, tip-associated adhesin PilY1 | PilY1 | 17.92 | 1.65 | 2.15 | 0.78 |
| AOLE_03010 | Tfp pilus assembly protein FimT | FimT | 3.91 | 2.97 | 2.12 | -1.94 |
| AOLE_06715 | P pilus assembly protein, chaperone PapD | FimC | 8.53 | 3.42 | 1.14 | -1.77 |
| AOLE_06720 | P pilus assembly protein, porin PapC | FimD | 28.10 | 2.30 | 2.57 | -1.62 |
| AOLE_07045 | P pilus assembly protein, chaperone PapD | FimC | 30.53 | 6.09 | 1.22 | 1.65 |
| AOLE_09765 | P pilus assembly protein, pilin FimA | FimA | 1.07 | 1.99 | 0.96 | 0.80 |
| AOLE_09770 | P pilus assembly protein, chaperone PapD | FimC | -1.53 | 5.73 | 1.30 | -1.68 |
| AOLE_09775 | P pilus assembly protein, porin PapC | FimD | 1.20 | 1.62 | 0.75 | 0.62 |
| AOLE_10830 | Type II secretory pathway, pseudopilin PulG | PulG | 2.89 | 1.73 | 0.71 | 1.23 |
| AOLE_11115 | P pilus assembly protein, pilin FimA | FimA | 2.15 | 27.84 | 2.03 | 1.00 |
| AOLE_11120 | P pilus assembly protein, chaperone PapD | FimC | 12.33 | 3.20 | 1.06 | -3.18 |
| AOLE_11130 | P pilus assembly protein, pilin FimA | FimA | 12.57 | 4.11 | 3.26 | 1.31 |
| AOLE_15225 | Tfp pilus assembly protein, pilus retraction ATPase PilT | PilT | 13.50 | 2.06 | 1.46 | 1.05 |
| AOLE_16925 | Tfp pilus assembly protein PilF | PilF | 1.67 | 1.49 | 0.46 | 1.14 |
| AOLE_17580 | Type II secretory pathway, pseudopilin PulG | PulG | 3.52 | 2.22 | 1.01 | -1.38 |
| AOLE_17795 | Type II secretory pathway, prepilin signal peptidase PulO and related peptidases | PulO | 4.85 | 1.71 | 1.31 | 1.16 |

**Table S3. Fimbriae/pili related gene expression profiles by different class antibiotics.**
