# Supplementary material for: Global Transcriptome and Physiological Responses of Acinetobacter oleivorans DR1 Exposed to Distinct Classes of Antibiotics
Source: PLoS One. 2014 Oct 17;9(10):e110215. doi: 10.1371/journal.pone.0110215 (PMC4201530; doi:10.1371/journal.pone.0110215)
Supplement: Table S4 — The feature of small RNA genes in A. oleivorans DR1. (DOCX) [file pone.0110215.s010.docx]

**Table S4. The feature of small RNA genes in *A. oleivorans* DR1**

|  | **Locus** | **Nucleotide location** | **Nucleotide length (bp)** | **GC percentage (%)** | **Species with the closest genome** |
| --- | --- | --- | --- | --- | --- |
| **Small RNA candidate 1** | AOLE14160-AOLE14165 | 3027649…3027816 | 168 | 50.59 | *Acinetobacter baumannii* |
| **Small RNA candidate 2** | AOLE15150-AOLE15155 | 3224850…3225198 | 349 | 48.42 | *Acinetobacter baumannii* |
| **Small RNA candidate 3** | AOLE15455-AOLE15460 | 3292748…3293091 | 344 | 56.68 | *Acinetobacter calcoaceticus* |
