# Supplementary material for: Global Transcriptome and Physiological Responses of Acinetobacter oleivorans DR1 Exposed to Distinct Classes of Antibiotics
Source: PLoS One. 2014 Oct 17;9(10):e110215. doi: 10.1371/journal.pone.0110215 (PMC4201530; doi:10.1371/journal.pone.0110215)
Supplement: Table S5 — Bacterial strains, plasmid and oligonucleotides sequence used in this study. (DOCX) [file pone.0110215.s011.docx]

**Table S5. Bacterial strains, plasmid and oligonucleotides sequence used in this study.**

| Bacterial strains/ Oligonucleotides | Description | Reference |
| --- | --- | --- |
| Strains and palsmid |  |  |
| *E. coli* GC 4468 | Reference strain of rifampicin mutagenesis | This study |
| *A. baumannii* ATCC17978 | Reference strain of rifampicin mutagenesis | This study |
| *A. oleivorans* DR1 | Wild-type, non-naphthalene degrader, diesel oil degrader | This study |
| *A. oleivorans* DR1(pRKprecA*::gfp*) | Insertion of pRk415-precA::gfp in *A.oleivorans* DR1 | This study |
| pRK415 | Tetracycline resistance gene, Broad-host-range vector | This study |
| Oligonucleotides |  |  |
| 16s rRNA-341F | 5’-CCT ACG GGA GGC AGC AG-3’ | Watanabe et al. |
| 16s rRNA-534R | 3’-ATT ACC GCG GCT GCT GGC A-5’ | Watanabe et al. |
| AOLE_00910-qPCR_FN | 5’-ATC TGG GCG TGG TGA TTG AG-3’ | This study |
| AOLE_00910-qPCR_RN | 3’-TTC GTG CAG CCG ACC ATA TT-5’ | This study |
| AOLE_01905-qPCR_FN | 5’-GGC AGA CCA CCC AAA TGG TA-3’ | This study |
| AOLE_01905-qPCR_RN | 3’-AGA ACC CAC TGC TGG CAA AT-5’ | This study |
| AOLE_07635-qPCR_FT | 5’-CGA TGG ATT AAT TGC CCA TCT TTG A-3’ | This study |
| AOLE_07635-qPCR_RT | 3’-TGT CCA CCG TGC CAC AAT AG-5’ | This study |
| AOLE_08415-qPCR_FT | 5’-CAT CGG TTG GGC GAT GGA TA-3’ | This study |
| AOLE_08415-qPCR_RT | 3’-GCA TTG AAG ATT GCG CAC CA-5’ | This study |
| AOLE_09285-qPCR_FA | 5’-GCT TTT CAC CAG CTT CAG GC-3’ | This study |
| AOLE_09285-qPCR_RA | 3’-TTA GGT CAA CGG TGG CAC TC-5’ | This study |
| AOLE_09770-qPCR_FK | 5’-TTC GGA TGC TCG CGA AGT TA-3’ | This study |
| AOLE_09770-qPCR_RK | 3’-AGC TGT TGG CAG AGC AGT AA-5’ | This study |
| AOLE_09790-qPCR_FA | 5’-ATT TGC CCA GCT TTC GCT TG-3’ | This study |
| AOLE_09790-qPCR_RA | 3’-TGG ATG TGG TGT GAC GAC TG-5’ | This study |
| AOLE_11145-qPCR_F | 5’-AGG AGC CGT TGA AGC CAA AT-3’ | This study |
| AOLE_11145-qPCR_R | 3’-AAA ATT GCG TGT GCT CGG TG-5’ | This study |
| AOLE_12135-qPCR_F | 5’-TGC TAC CAA TCG GAT ACT GAC T-3’ | This study |
| AOLE_12135-qPCR_R | 3’-GGG GTT CCT GTT TCA ACC CT-5’ | This study |
| AOLE_13500-qPCR_FA | 5’-TGA AGC AGC GCG TTT ACA GT-3’ | This study |
| AOLE_13500-qPCR_RA | 3’-CTC GCC TTG CAT CAT TTT AGC-5’ | This study |
| AOLE_14800-qPCR_F | 5’-GCA TCT GGT CTT CGG GTG AA-3’ | This study |
| AOLE_14800-qPCR_R | 3’-CGC GTG ATG AGT TAT TCG GC-5’ | This study |
| AOLE_14185-qPCR_FK | 5’-AGT AGG CCC TCC TGG TGT AG-3’ | This study |
| AOLE_14185-qPCR_RK | 3’-TGG GTT CTT CAC GCC AAC TT-5’ | This study |
| AOLE_14840-qPCR_FK | 5’-GCA CGC CAA GAA TTA GCT GG-3’ | This study |
| AOLE_14840-qPCR_RK | 3’-ACG CTC ATA AGC ATT CAC TAC G-5’ | This study |
| AOLE_16860-qPCR_F | 5’-ACC CGC ACA TGC TGT CTT TA-3’ | This study |
| AOLE_16860-qPCR_R | 3’-GAT GGT CAA CAC TGC TGG GA-5’ | This study |
| AOLE_17345-qPCR_FT | 5’-CTC ACG GCT TGA ACC ACA AC-3’ | This study |
| AOLE_17345-qPCR_RT | 3’-CAA ACG TAC TGG TTT TGG CGA-5’ | This study |
| AOLE_17410-qPCR_FN | 5’-GCG GTA AAT TGT TCG CCA CA-3’ | This study |
| AOLE_17410-qPCR_RN | 3’-GGG AAG CTG CTG GTG TTG AT-5’ | This study |
| pRKprecA-gfp-F | 5’-CGC **GGT ACC** TTT GTG CCA ATT TCC CAG CG-3’ | This study |
| pRKprecA-gfp-R | 3’-CGC **GGA TCC** ATG AGT GAT GTG ACT CCC GC-5’ | This study |
| pRK gfp-F | 5’-CGC **GGA TCC** TGA GAT CCT AAA AAT CTA TCA-3’ | Hong et al. |
| pRK gfp-R | 3’-CGC **GAA TTC** TTA TTT AGC GCT CTT TAA TAC-5’ | Hong et al. |
| GFP-F | 5’TTG TTG AAT TAG ATG GCG ATG TTA-3’ | Hong et al. |
| GFP-R | 3’-TTT GGA AAG GGC AGA TTG TGT-5’ | Hong et al. |
| 30F-F | 5’-GTCACGTGCTGCA**F**ACGACGTGCTGAGCCT-3’ | This study |
| 30F-R | 3’-CAGTGCACGACGTGAGCTGCACGACTCGGA-5’ | This study |
| U30-F | 5’-GTCACGTGCTGCA**U**ACGACGTGCTGAGCCTC-3’ | This study |
| U30-R | 3’-CAGTGCACGACGTGTGCTGCACGACTCGGAG-5’ | This study |
| OxoG-F | 5’-GTCACGTGCTGCA**8**ACGACGTGCTGAGCCTC-3’ | This study |
| OxoG-R | 3’-CAGTGCACGACGTCTGCTGCACGACTCGGAG-5’ | This study |
| sRNA1 F | 5’-CTC ATA CGC TCA AGC GAC CT-3’ | This study |
| sRNA1 R | 3’-CCG CCT CAT TCG TGA GGT AA-5’ | This study |
| sRNA1 F | 5’-TAA CAC CAA GTG ACC CGA CG-3’ | This study |
| sRNA1 R | 3’-AGC TGC CTA AGG GCA GTT TG-5’ | This study |
| sRNA1 F | 5’-TTC GCC AGC GGG TCT ATT C-3’ | This study |
| sRNA1 R | 3’-GCT GCA TGT GTC GTT ACC CT-5’ | This study |

F = tetrahydrofuran (abasic site analog), U = deoxyuridine 8 = 8-oxoguanine

Hong H, Park W (2014) TetR repressor-based bioreporters for the detection of doxycycline using *Escherichia coli* and *Acinetobacter oleivorans.* Microb Ecol 67: 369-379.

Watanabe K, Kodama Y, Harayama S (2001) Design and evaluation of PCR primers to amplify bacterial 16S ribosomal DNA fragments used for community fingerprinting. J Microbiol Methods 44: 253e262.
